# Supplementary material for: Vibrationally-dependent molecular dynamics in mutual neutralisation reactions of molecular oxygen ions
Source: Nat Commun. 2025 Sep 26;16:8528. doi: 10.1038/s41467-025-64198-0 (PMC12474973; doi:10.1038/s41467-025-64198-0)
Supplement: Supplementary file 1 — Supplementary Information [file 41467_2025_64198_MOESM1_ESM.pdf]

Supplementary Information: Vibrationally-dependent molecular dynamics in mutual neutralisation reactions of molecular oxygen ions

Mathias Poline[1], Arnaud Dochain[1,2], Stefan Rosén[1], MingChao Ji[1], Henrik Cederquist[1], Henning Zettergren[1], Henning T. Schmidt[1], Mats Larsson[1], Shaun G. Ard[3], Nicholas S. Shuman[3], Albert A. Viggiano[3], Richard D. Thomas[1*]

[1]Department of Physics, Stockholm University, SE-106 91, Stockholm, Sweden
[2]Institute of Condensed Matter and Nanosciences, Université Catholique de Louvain, Louvain-la-Neuve, B-1348, Belgium
[3]Space Vehicles Directorate, Air Force Research Laboratory, Kirtland AFB, Albuquerque, 87117, New Mexico, USA
*rdt@fysik.su.se

Supplementary Methods

In order to evaluate the data, appropriate methods must be used to determine the momentum vectors and energies of the products, and Monte Carlo simulations of the particles trajectories must be implemented. The reaction can in principle lead to both two- and three-body products, and these are treated independently.

Supplementary Note 1.1: Two-body analysis

In a two-body scenario, i.e reaction (1) in the main text, both products receives a fixed amount of energy, and the calculation of their final kinetic energy and momentum vectors is straightforward (see, e.g. (1; 2)). The resultant experimental two-body $E_{\mathrm{K_f}}$ spectrum for the MN of $^{16,16}O_2^+$ with O^- is shown in Suppl. Fig. 1, plotted as filled blue circles where the error bars are the standard deviations. Events with centre-of-mass outside a 5 mm radius from the average centre-of-mass of the two beams are filtered out, to eliminate signals originating from collisions with the residual gas. As the MCP detection efficiency is not 100%, three-body events in which one particle is not detected, but which still satisfies centre-of-mass filtering, contribute to the two-body $E_{\mathrm{K_f}}$ data.

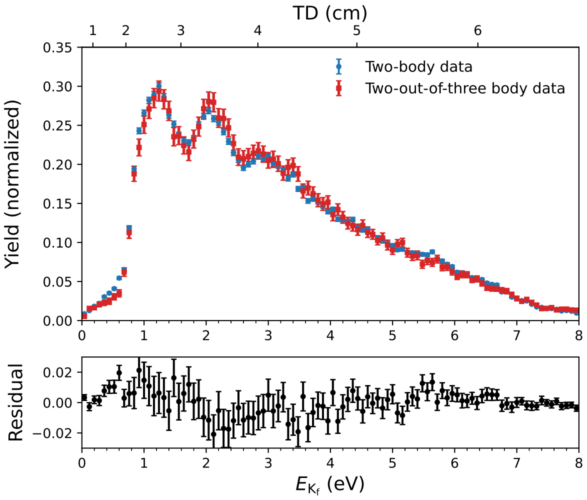

Supplementary Fig. 1 Kinetic energy release, $E_{\mathrm{K_f}}$, distributions for two-body coincidence data. The data indicated by the filled blue circles shows the measured two-body data, and the red filled circles the contribution from three-body data where one neutral product is randomly removed. The error bars are the standard deviations.

This contribution can be evaluated by randomly selecting two products out of the three from the three-body data presented in the main article in Fig. 1, and these data are indicated by the red filled circles in Suppl. Fig. 1 here. Comparison of these two data sets allows the true, two-body $E_{\mathrm{K_f}}$ spectra to be determined.

While analysis of the two-body $E_{\mathrm{K_f}}$ data plotted in Suppl. Fig. 1 reveals distinct structures, the exact same features are present in the random selection of two products from the three-body signal. The structure likely arises due to the intrinsic dynamics in the three-body reaction, which has one fragment taking a constant fraction of the energy in different channels. Examination of the residuals shown under the main plot reveals some fluctuations at low $E_{\mathrm{K_f}}$ values. Similarly as for the three-particle data, due to the small separations, we assign this as likely arising from false coincidences due to the high flux of neutrals striking the centre of the detector from the ion beams, and the limited ability of the detection system to accurately distinguish one-or-two particle hits at such small separations given the practical limitations on the sizes of the individual spots. However, given the statistical uncertainties, we do not rule out a possible small contribution from two-body channels.

Supplementary Note 1.2: Three-body analysis

For three products, energy and momentum conservation allows a wide range of energy sharing, and the work of U. Müller, P. C. Cosby and co-workers, who derived a general model to describe the three-body fragmentation of H_3, is used as a basis for evaluation of these data (3; 4). The fraction of the available energy, E, that each particle carry can be described by two parameters a and b according to:

$$E_1 = aE, \qquad E_2 = bE, \qquad E_3 = cE, \tag{1}$$

where E_{i} are the kinetic energies of each one of the three products, and $c = 1 - a - b$. The momentum vectors can always be described to lie in an arbitrary plane, and can be defined as:

$$\mathbf{p}_1 = (p_{1\mathrm{x}}, 0, 0)\,, \quad \mathbf{p}_2 = (p_{2\mathrm{x}}, p_y, 0)\,, \quad \mathbf{p}_3 = (-(p_{1\mathrm{x}} + p_{2\mathrm{x}}), -p_{\mathrm{y}}, 0)\,, \tag{2}$$

where $p_{1\mathrm{x}}$, $p_{2\mathrm{x}}$, and p_{y} are defined as

$$p_{1\mathrm{x}} = \sqrt{2aEm}, \quad p_{2\mathrm{x}} = \frac{(1-2b-2a)Em}{p_{1\mathrm{x}}}, \quad p_{\mathrm{y}} = \sqrt{2mbE - p_{2\mathrm{x}}^2}. \tag{3}$$

From energy and momentum conservation, the limits of a and b can be determined. Since the reaction can take place in a plane with any orientation in space, the vectors in the plane are then rotated randomly along both axes using rotation matrices. The displacement of each fragment from the centre of mass due to the kinetic energy released, E, can then be derived starting with the following relation:

$$\mathbf{r_i} = \frac{L}{v}\frac{\vec{p}_i}{m}, \tag{4}$$

where L is the distance to the interaction region, and v is the average velocity of the two ion beams. The total displacement (TD) of the fragments on the detector is then determined by:

$$\mathrm{TD} = \sqrt{\sum_{i=1}^{3} \mathbf{r_i}^2} \tag{5}$$

From this, the kinetic energy released in the reaction can be reconstructed:

$$E = \frac{1}{2}\, m \left(\frac{v \cdot \mathrm{TD}}{L}\right)^2 . \tag{6}$$

This equation is exact, if the distance from the point of interaction, L, to the detector would be known with high precision. However, the reaction can take place at any point along the biased interaction region and the average distance is used to compute E, which limits the energy resolution, and results in a broadening of the peaks. An additional broadening is present due to the internal energy in the parent ions, which for three-body break-up is completely converted into kinetic energy in the products as:

$$E_{\mathrm{K_f}} = E_{\mathrm{K}} + E_{\mathrm{c.m.}} + E_{\mathrm{rot}} + E_{\mathrm{vib}}, \tag{7}$$

where $E_{\rm rot}$ is the initial rotational energy, $E_{\rm vib}$ the initial vibrational energy, and $E_{\rm c.m.}$ the collision energy. These three quantities have different effects on the final kinetic energy distribution: The vibrational energy mainly results in a tail to higher $E_{\rm K_f}$ values, whereas the rotational and collision energy distribution result in both a broadening and a shift of the $E_{\rm K_f}$ distributions towards higher values. This shift can be observed in the spectra, as the distributions peaks at higher values than the expected values (reactions (1 - 5) in the main text).

In the simulations, the broadening due to the length of the interaction region is taken into account by assuming a uniform distribution of events at $L = 1.78 \pm 0.12$ m, corresponding to the size of the biased region. This broadening scales linearly with $E_{\rm K_f}$, and ranges between 0.5 and 1.5 eV for the channels considered here. The additional broadening effects are taken into account by simulating a Boltzmann distribution of vibrational and rotational energy, and an assumed collision energy of $E_{\rm c.m.}$= 0.1±0.05 eV (based on previous measurements at DESIREE). A rovibrational energy distribution of 3000 K was found to best describe the data for the $^{16,16}O_2^+$ ions, and 1000 K vibrational (3000 K rotational) energy distribution for the $^{18,16}O_2^+$ ions at later storage times of 30-60 seconds. The vertical bars presented in the fits to symbolise the positions of the vibrational levels, were shifted by both the average rotational and collision energy to reflect this. The fits are highly sensitive to these parameters and we estimate the uncertainty in the temperatures given to be of ± 500 K, as indicated in the main text.

Supplementary Note 1.3: Three-body reaction dynamics

The dynamics of the reaction can then be evaluated based on the energy partitioning among the fragments, i.e., parameters a and b in equation (1). For example, in the case of a linear break-up, $a = 0$, and the energy is shared equally between the two other fragments. This would be visible in a Dalitz plot, which represents the three energy fractions in a single two-dimensional plot. These are defined by the generalized coordinates (3; 4):

$$\eta_1 = \frac{E_1 - E_2}{\sqrt{3}E} \quad (8a)$$

$$\eta_2 = \frac{2E_3 - E_2 - E_1}{3E} \quad (8b)$$

which, due to energy and momentum conservation, confines all allowed geometries into a circle of radius 1/3.

Supplementary Note 1.4: The free-rotor model in two-step reactions

In the case of a dissociation via an intermediate state, the reaction can be described as follows:

$$O_2^+ + O^- \rightarrow O_2^* + O + E_{\rm K_i} \rightarrow O + O + O + E_{\rm K_f}, \quad (9)$$

i.e, there is an intermediate kinetic energy release $E_{\rm K_i}$ which leaves O_2 in an excited state, and there is a subsequent, final kinetic energy release, $E_{\rm K_f}$, for the complete process which includes the kinetic energy released in the dissociation of O_2^*. Within the free-rotor model (5; 6), we assume that the neutralised anion no longer participates in the reaction after the initial electron-transfer step, i.e. no further energy is then exchanged between O and O_2^*. The fraction of the available energy taken by the neutralised anion is then determined by the kinetic energy, $E_{\rm K_i}$, released as the intermediate state O_2^* is formed. This fraction defines the value of parameter a in equation (1) for a two-step process of the kind discussed here. As seen from the centre-of-mass of O_2^*, the kinetic energies of both O-atoms must be the same, and their momenta opposite. However, in the centre-of-mass of all three atoms, the length of the momentum vectors may differ, and thus we measure different kinetic energies in the lab frame and in the centre-of-mass of the complete system. To reflect this, the remaining energy is randomly distributed between the two other fragments, b and c. This effectively results in a line in the Dalitz plot, as can be inferred from the Dalitz coordinates.

From conservation of energy and momentum, the kinetic energy released when the intermediate state

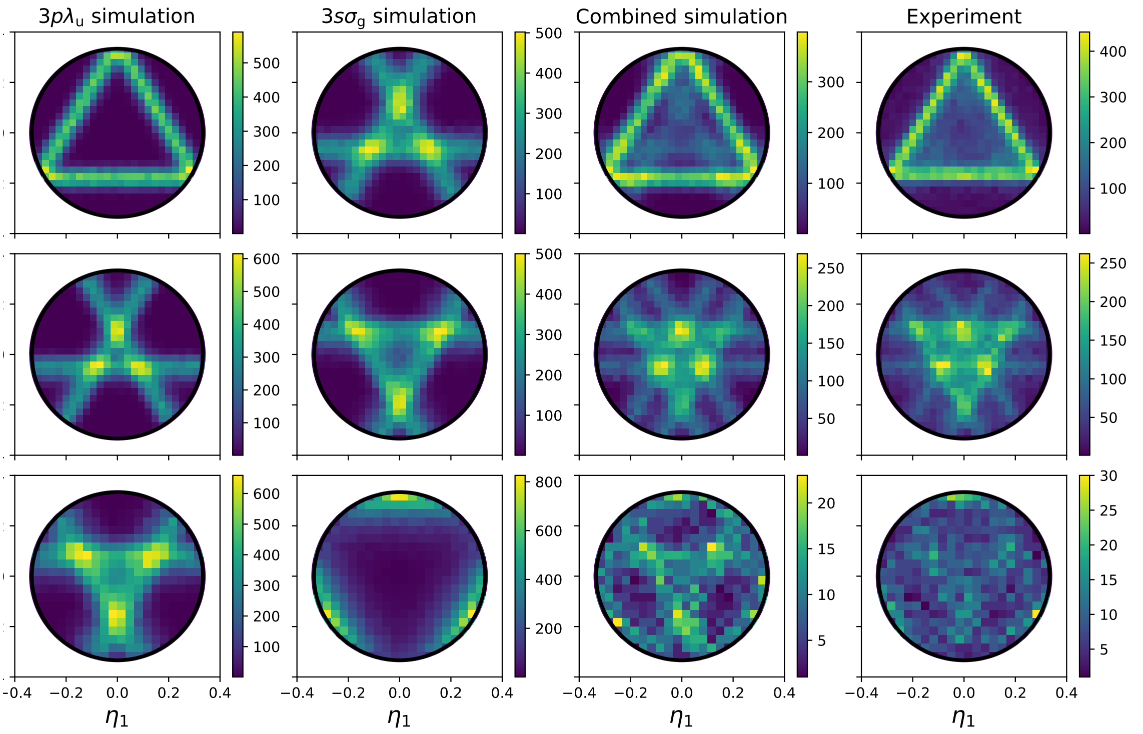

Supplementary Fig. 2 Dalitz plots for the three observed final product channels. Plots on the top, middle, and bottom rows correspond to the reaction channels (2) O(^{3}P)+O(^{3}P)+O(^{3}P), (3) O(^{1}D)+O(^{3}P)+O(^{3}P), and (4) O(^{1}D)+O(^{1}D)+O(^{3}P), respectively. Experimental data are shown in the far right-hand column. The plots in the first two columns show results from simulations of the $3p\lambda_u$ and $3s\sigma_g$ intermediate states, with the relative contribution of these to the observed experimental data shown in the third column.

is formed is thus determined by the kinetic energy, E_{O}, of the neutralised anion:

$$E_{\mathrm{K_i}} = \frac{3m_{\mathrm{O}}}{2m_{\mathrm{O}}} E_{\mathrm{O}} \tag{10}$$

This result is exact provided that the neutralised atomic anion can be correctly identified among the three separating O-atoms. Here, the three particles have the same mass, and specific selections are applied to aid the particular identification, based on the observed features in the Dalitz plots (see Fig. 2 in the main text). For the O(^{3}P)+O(^{3}P)+O(^{3}P) channel (2), the kinetic energy release in the first step is much smaller than the kinetic energy release in the second step, and the neutralised oxygen anion most often takes on the smallest amount of energy. The opposite is true for the O(^{1}D)+O(^{1}D)+O(^{3}P) channel (4), and, in this case, the oxygen with the largest energy instead is selected. For the O(^{1}D)+O(^{3}P)+O(^{3}P) channel (3), no appropriate selection could be made, and the neutralised oxygen anion is chosen at random. This imperfect selection results in an apparent background associated with the $E_{\mathrm{K_i}}$ spectra. The same selection is implemented in the simulation to reproduce this background, and make an appropriate fit.

The fit can then be used to reproduce the experimental Dalitz spectra. Separate simulations are performed for the $3s\sigma_g$ and $3p\lambda_u$ intermediate states, and then combined according to the values obtained from the fit. Supplementary Fig. 2 shows the results of this analysis. As can be seen, the combined simulated Dalitz plots are very similar to the experimental ones, indicating that the fitting

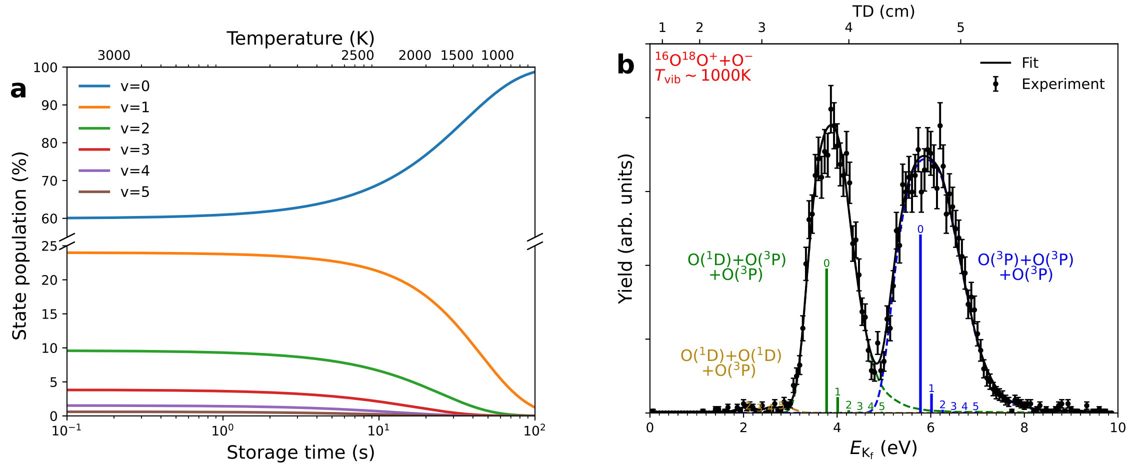

Supplementary Fig. 3 Storage-time dependent vibrational state distributions and coincident three-body total kinetic energy release, E_{K_f}, distributions. **a** Calculated vibrational state distributions in $^{16,18}O_2^+$ as function of ion storage time with a starting temperature of 3000 K. DESIREE's $\approx$ 20 K radiation field (7; 8), and $\Delta v > 1$ transitions, are neglected; **b** Coincident three-body total kinetic energy release, E_{K_f}, and Total Displacement, TD, distributions from MN of $^{16,18}O_2^+$ + O^- at storage times 30-60 s. Experimental data are plotted as filled circles and the error bars are the standard deviations.

procedure was successful.

Supplementary Note 1.5: Vibrational cooling

As discussed in the main text, the vibrational lifetimes for the mixed $^{16,18}O_2^+$ isotopologue have been calculated to be on the order of seconds (9). Using the data from ref. (9), the vibrational population as a function of storage time is shown in Suppl. Fig. 3a, and, after 60 s, a 95% ground-state population is expected. After about 30 s of storage, complete depletion of the higher vibrational states ($v \geq 4$) is expected, and, for both experimental and technical reasons, signals are analysed for the first 0-5 s and the last 30-60 s of storage. The latter are the data plotted in Suppl. Fig. 3b.

To obtain the branching fractions into the various channels for the later storage time data, a similar fitting procedure is undertaken as for the early data described in the main text. The fit of simulated distributions for the $^{16,18}O_2^+$ data is shown in Suppl. Fig. 3b as the solid black line. Here, the internal energy of the O_2^+ ions is described by a 1000 K vibrational Boltzmann distribution (while keeping the initial, unchanged 3000 K rotational population). As before, this describes the prominent experimental features well. What remains of the smaller channel is again only described by non-Boltzmann, individually-fit contributions.

Supplementary References

[1] Poline, M. *et al.* Mutual neutralisation of O^+ with O^-: investigation of the role of metastable ions in a combined experimental and theoretical study. *Phys. Chem. Chem. Phys.* **23**, 24607–24616 (2021).

[2] Poline, M. *et al.* Storage-ring study of the mutual neutralization of N^+ with O^-. *Phys. Rev. A* **105**, 062825 (2022).

[3] Müller, U., Eckert, Th., Braun, M. & Helm, H. Fragment Correlation in the Three-Body Breakup of Triatomic Hydrogen. *Phys. Rev. Lett.* **83**, 2718–2721 (1999).

[4] Müller, U. & Cosby, P. C. Three-body decay of the $3s^2A_1'(N = 1, K = 0)$ and $3d^2E''(N = 1, G = 0, R = 1)$ Rydberg states of the triatomic hydrogen molecule H_3. *Phys. Rev. A* **59**, 3632–3642 (1999).

[5] Hishikawa, A., Hasegawa, H. & Yamanouchi, K. Sequential three-body Coulomb explosion of CS_2 in intense laser fields appearing in momentum correlation map. *Chemical Physics Letters* **361**, 245–250 (2002).

[6] Poline, M. *et al.* Mutual Neutralization of NO^+ with O^-. *Phys. Rev. Lett.* **132**, 023001 (2024).

[7] Schmidt, H. T. *et al.* Rotationally Cold OH^- Ions in the Cryogenic Electrostatic Ion-Beam Storage Ring DESIREE. *Phys. Rev. Lett.* **119**, 073001 (2017).

[8] Schmidt, H. T. *et al.* Erratum: Rotationally Cold OH^- Ions in the Cryogenic Electrostatic Ion-Beam Storage Ring DESIREE [Phys. Rev. Lett. 119, 073001 (2017)]. *Phys. Rev. Lett.* **121**, 079901 (2018).

[9] Amitay, Z., Zajfman, D. & Forck, P. Rotational and vibrational lifetime of isotopically asymmetrized homonuclear diatomic molecular ions. *Phys. Rev. A* **50**, 2304–2308 (1994).
